# Kisspeptin treatment reverses high prolactin levels and improves gonadal function in hypothyroid male rats

**DOI:** 10.1038/s41598-023-44056-z

**Published:** 2023-10-05

**Authors:** Luciano Cardoso Santos, Jeane Martinha dos Anjos Cordeiro, Larissa da Silva Santana, Erikles Macêdo Barbosa, Bianca Reis Santos, Letícia Dias Mendonça, Maria Clara da Silva Galrão Cunha, William Morais Machado, Larissa Rodrigues Santana, Maíra Guimarães Kersul, Patrícia Costa Henriques, Roberta Araújo Lopes, Paola Pereira das Neves Snoeck, Raphael Escorsim Szawka, Juneo Freitas Silva

**Affiliations:** 1 Centro de Microscopia Eletronica, Departamento de Ciencias Biologicas, Universidade Estadual de Santa Cruz, Campus Soane Nazare de Andrade, Ilheus, 45662-900 Brazil; 2 Laboratorio de Reprodução Animal, Departamento de Ciencias Agrarias e Ambientais, Universidade Estadual de Santa Cruz, Campus Soane Nazare de Andrade, Ilheus, 45662-900 Brazil; 3 Laboratorio de Endocrinologia e Metabolismo, Departamento de Fisiologia e Biofisica, Universidade Federal de Minas Gerais, Belo Horizonte, 31270-901 Brazil

**Keywords:** Systems biology, Endocrinology, Urology

## Abstract

We evaluated whether the administration of kisspeptin-10 (Kp10) is capable of restoring gonadal function in hypothyroid male rats. Hypothyroidism was induced with 6-propyl-2-thiouracil (PTU) for three months. In the last month, half of the hypothyroid animals were treated with Kp10. Hypothyroidism reduced testicular and sex gland mass, decreased the proliferation of the seminiferous epithelium, and compromised sperm morphology, motility, and vigor. A decrease in plasma LH and testosterone levels and an increase in prolactin secretion were observed in the hypothyroid rats. Hypothyroidism reduced Kiss1 and Kiss1r protein and gene expression and *Star* and *Cyp11a1* mRNA levels in the testis. Furthermore, it reduced *Lhb*, *Prl*, and *Drd2* and increased *Tshb* and *Gnrhr* expression in the pituitary. In the hypothalamus, hypothyroidism increased *Pdyn* and *Kiss1r* while reducing *Gnrh1*. Kp10 treatment in hypothyroid rats restored testicular and seminal vesicle morphology, improved sperm morphology and motility, reversed high prolactin levels, and increased LH and testosterone levels. In addition, Kp10 increased testicular expression of *Kiss1*, *Kiss1r, Fshr*, and *Nr5a1* and pituitary *Kiss1* expression. Our findings describe the inhibitory effects of hypothyroidism on the male gonadal axis and sperm quality and demonstrate that Kp10 treatment reverses high prolactin levels and improves gonadal function and sperm quality in hypothyroid rats.

## Introduction

Between 9 and 25% of couples worldwide suffer from infertility and the male factor is responsible for approximately half of these cases^1^. Sperm quality is an important indicator of male fertility and studies have been showing a downward trend in semen quality all over the world^2^. One of the causes of this decline is hypothyroidism, an important endocrinopathy that affects sperm quality^3–5^ and, consequently, fertility in men^6,7^.

Hypothyroidism in males delays puberty, causes erectile dysfunction^8,9^, and reduces testicular mass^7–9^, gonadotropin-releasing hormone (GnRH) biosynthesis, and the plasma levels of follicle stimulating hormone (FSH)^10^, luteinizing hormone (LH)^10^ and testosterone^10,11^. Since the effects of propylthiouracil (PTU)-induced hypothyroidism upon male fertility and reproductive function are similar to those produced by other models of hypothyroidism, as that induced by methimazole (MMI)^12,13^, and levothyroxine (T_4_) treatment is able to revert them^12^, we may be fairly sure that these reproductive outcomes are due to the hypothyroid state induced by PTU and not to any direct effects of PTU. Furthermore, hypothyroidism causes hyperprolactinemia^10^, a condition that has also been considered as a cause of infertility in men, with a prevalence of 11% in hypothyroid men^14^. Moreover, hypothyroidism affects the differentiation of Sertoli and Leydig cells^7,9^, which is also involved in testicular dysfunction caused by thyroid hypofunction.

In addition to the hormonal changes and structural alterations in the gonad, hypothyroidism can have a direct effect on the hypothalamus-pituitary-gonadal (HPG) axis^9^. The presence of thyroid hormone alpha receptor (Thrα) has been demonstrated not only in the GnRH neurons of rodents^15^ but also in the kisspeptin neurons of the sheep hypothalamus^16^. This suggests that thyroid hormone deficiency can directly affect the activity of these neuronal populations.

The control of the pulsatile release of GnRH in the pituitary portal blood and, consequently, the gonadal function is dependent on the release of kisspeptin by neurons in the arcuate nucleus (ARC) of the hypothalamus^17^. In this region, kisspeptin neurons co-express neurokinin-B (NKB) and dynorphin (KNDy neurons), which exert stimulatory and inhibitory effects on kisspeptin release, respectively, resulting in a pulsatile drive to the GnRH secretion^18,19^. We recently demonstrated that hypothyroidism in female rats reduces kisspeptin and *Nkb* expression in KNDy neurons, and the treatment with kisspeptin-10 (Kp10) restores ovarian cyclicity and plasma LH levels in these animals^20^. However, there is still little knowledge about alterations in KNDy neuronal function in male rats with hypothyroidism. Furthermore, previous studies have shown the therapeutic potential of kisspeptin in males with testicular dysfunction. Accordingly, the kisspeptin receptor (kiss1r) agonists have been found to restore testosterone levels in Rhesus monkeys with the HPG axis blockade caused by cortisol^21^ and underweight male rats dosed with an anti-obesogenic compound^22^.

However, not only is hypothalamic regulation of KNDy neurons necessary for a normal reproductive function, but studies also suggest that the presence of the kisspeptin/Kiss1r system in the testis may influence gonadal function^23,24^. The expression of Kiss1 gene and Kiss1r has been described mainly in the Leydig cells mice^25,26^, non-human primates^27^, and horses^28^, while in goats they have also been found in the Sertoli cells and seminiferous epithelium^29,30^. *In vitro* studies have shown that kisspeptin stimulates testosterone production by the Leydig cells in goats as well as displays a positive self-regulation that stimulates *Kiss1* and *Kiss1r* gene expression in these cells^29^. Gan et al.^31^ also recently demonstrated *in vitro* that an increased Kiss1 and Kiss1r expression in Sertoli cells protects against the apoptosis caused by hyperglycemia. However, there is still no information on whether hypothyroidism can affect the testicular expression of the Kiss1/kiss1r system and whether the treatment with kisspeptin could modulate the expression of this system.

In the present study, we investigated the HPG axis of male rats with hypothyroidism, the testicular expression of the Kiss1/Kiss1r system, and the therapeutic potential of Kp10 in the gonadal dysfunction of these animals. We demonstrate herein that hypothyroidism in male rats increased the hypothalamic expression of prodynorphin and compromised testicular Kiss1/Kiss1r expression, whereas the administration of Kp10 not only reversed the high PRL levels caused by hypothyroidism but also improved the gonadal axis, sperm morphology, and testicular expression of Kiss1 and Kiss1r. To the best of our knowledge, this is the first study to provide information on KNDy neurons and the testicular Kiss1/Kiss1r system in the gonadal dysfunction caused by hypothyroidism.

## Results

### Confirmation of hypothyroidism and mating test

**Figure 1:**
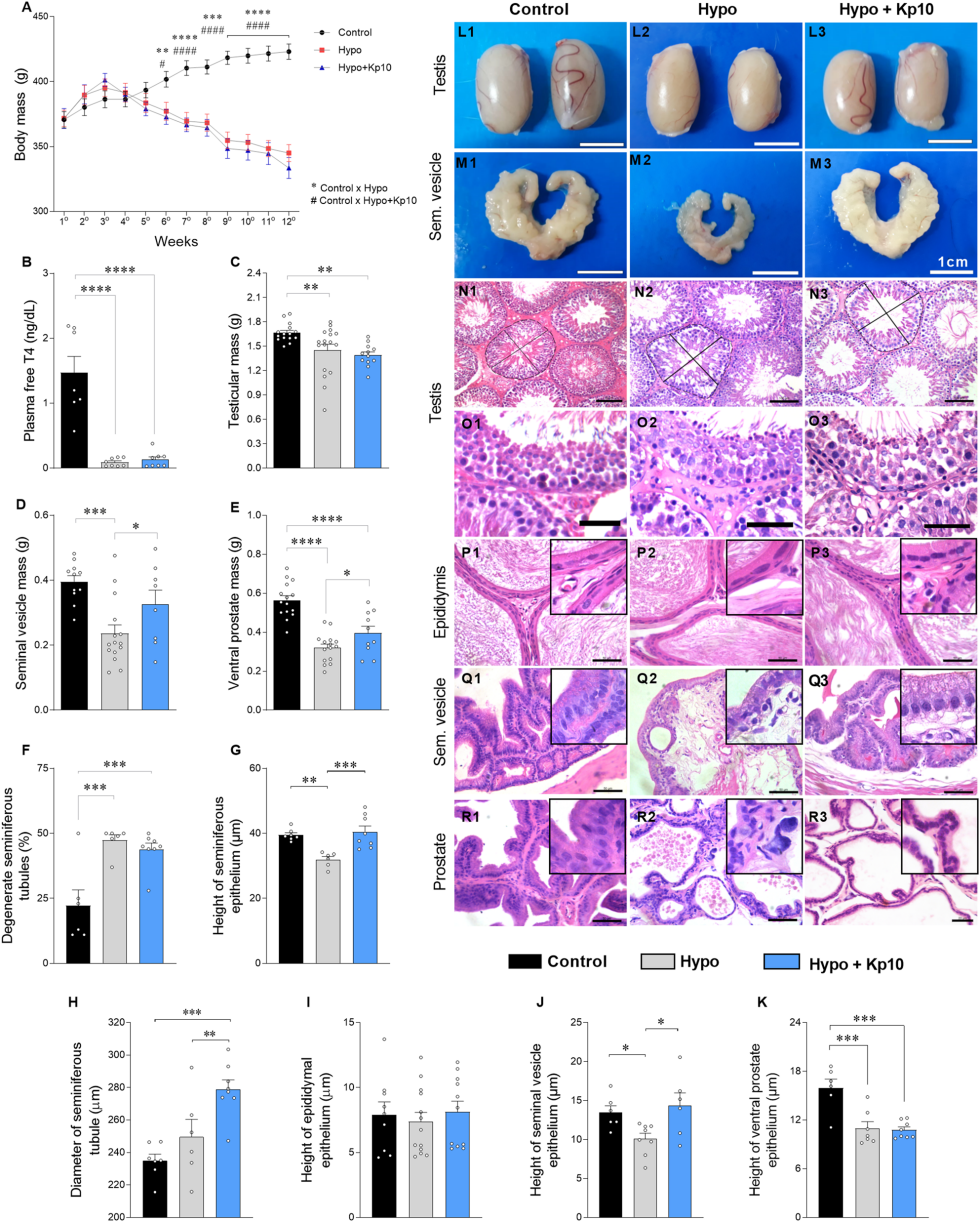
Confirmation of hypothyroidism and effects of kisspeptin-10 on genital tract morphology in hypothyroid rats. (**A**) Body mass of Control, Hypo and Hypo + Kp10 rats throughout the experimental period (n = 8–17; # = control × hypo; * = control × hypo + Kp10). (**B**) Free T_4_ levels (ng/dL) (n = 7–8). Testis mass (**C**), emptied seminal vesicle mass (**D**) and prostate mass (**E**) of rats in the Control, Hypo and Hypo + Kp10 groups (n = 8–17). (**F**) Percentage of degenerated seminiferous tubules in rats in the Control, Hypo and Hypo + Kp10 groups (mean ± SEM; n = 6–8). (**G**) Height of the seminiferous tubule epithelium of rats in the Control, Hypo and Hypo + Kp10 groups (n = 30 tubules/animal). (**H**) Tubular diameter of rats in the Control, Hypo and Hypo + Kp10 groups (n = 30 tubules/animal). (**I**–**K**) Height of epididymal epithelial (**I**), seminal vesicle (**J**) and ventral prostate (**K**) of rats in the Control, Hypo and Hypo + Kp10 groups (n = 50 epididymal regions/animal; 50 glandular acini/animal). (**L**,**M**) Images of the testis (**L**) and seminal vesicle (**M**) of rats in the Control (**L1**, **M1**), Hypo (**L2**, **M2**) and Hypo + Kp10 (**L3**, **M3**) groups; (**N**,**O**) Photomicrographs of seminiferous tubules from rats in the Control (**N1** and **O1**), Hypo (**N2** and **O2**) and Hypo + Kp10 (**N3** and **O3**) groups; arrows = apoptosis in basal cells and round spermatids. (**P**) Photomicrographs of the tail of the epididymis of rats in the Control (**P1**), Hypo (**P2**) and Hypo + Kp10 (**P3**) groups. (**Q**) Photomicrographs of the seminal vesicle of rats in the Control (**Q1**), Hypo (**Q2**) and Hypo + Kp10 (**Q3**) groups. (**R**) Photomicrographs of the ventral prostate of rats in the Control (**R1**), Hypo (**R2**) and Hypo + Kp10 (**R3**) groups. [Hematoxylin & Eosin; Bar scale: 1 cm (**L**,**M**), 50 µm (**N**; **O**; **P**–**R**)]. Captions: Hypo = hypothyroid; Hypo + Kp10 = hypothyroid treated with Kp10; *P < 0.05; **P < 0.01; ***P < 0.001; ****P < 0.0001.

The hypothyroid rats displayed a lower body mass than the control group after the sixth week of treatment with PTU (Fig. 1A) and reduced plasma free T_4_ levels (Fig. 1B; ****P < 0.0001), confirming the induction of hypothyroidism during the experimental period. To assess fertility, 13 to 15 animals from each group were individually housed with cycling female rats (1 male/1 female) for nine days prior to euthanasia. The estrous cycle of the female rats was monitored for a period of 15 days before copulation, and only female rats with regular cycles were mated (Supplementary Fig. S1 A–C). In all, 73% (11/15) of female rats kept with male rats from the control group had offspring (Supplementary Fig. S1 D), while the number of females that gave birth was significantly lower when mated with hypo (23%; 3/13; Supplementary Fig. S1 D; *P < 0.05) and hypo + Kp10 rats (21.4%; 3/14; Supplementary Fig. S1 D; **P < 0.01). Mean litter size did not vary between groups (Supplementary Fig. S1 E; SNK test; *P* > 0.05).

In half of the females in each group, the gestational diagnosis was also performed through vaginal cytology. Of these, approximately half of the female rats kept with rats from the hypo (3/5) or hypo + Kp10 (2/6) groups had mated. However, there was no significant difference in relation to the percentage of females that had confirmed copulation with rats in the control group (6/7) (Supplementary Fig. S1 F; *P* > 0.05).

To estimate a possible pregnancy loss rate, we subtracted the number of females that gave birth from the total number of females that had copulation confirmed by vaginal cytology (Supplementary Fig. S1 F). As a result, females that mated with male rats from the hypo group (2/3; 66.7%) showed a tendency towards a higher rate compared to the control females (0%; 0/6) (Supplementary Fig. S1 G; Fisher's exact test; *P* = 0.08). The body mass of the three-day-old pups did not differ between groups (Supplementary Fig. S1 H; SNK test; *P* > 0.05) and no macroscopic changes were observed (Supplementary Fig. S1 I–K). These data demonstrate that the treatment with Kp10 was not able to improve the low rate of birth and copulation caused by hypothyroidism in male rats.

### Kp10 treatment improves testicular and sex gland morphology in hypothyroid rats

In the evaluation of the genital tract (Fig. 1C–R), hypo and hypo + Kp10 groups exhibited a reduced testicular mass relative to control (Fig. 1C; **P < 0.01). Regarding the sex glands, hypo rats displayed a marked reduction in the mass of the empty seminal vesicle (Fig. 1D; ****P < 0.001) and ventral prostate (Fig. 1E; ****P < 0.0001) compared to control. The treatment with Kp10, in turn, increased the mass of these glands compared to the hypo group, with the mass of the seminal vesicle being similar to the control group (Fig. 1D; *P* > 0.05).

Histopathological and histomorphometric evaluations of the testes (Fig. 1L, N–O) showed that hypothyroidism caused mild to moderate multifocal tubular degeneration, with superficial and basal intraepithelial vacuolation in the seminiferous epithelium (Fig. 1N2–O2). The percentage of degenerated tubules was about three times higher in the hypo group compared to control (Fig. 1F; ***P < 0.0001). These changes were accompanied by a reduction in the height of the seminiferous epithelium compared to the control group (Fig. 1G; **P < 0.01), but there was no change in tubular diameter (Fig. 1H; *P* > 0.05). Although the percentage of degenerated tubules in the Kp10-treated animals did not differ from the hypo group (Fig. 1F; *P* > 0.05), the height of the seminiferous epithelium was greater (Fig. 1G; ***P < 0.001), being similar to the control group (Fig. 1G; *P* > 0.05). In addition, the tubular diameter was greater in the hypo + Kp10 group compared to control and hypo (Fig. 1H; ***P < 0.001; **P < 0.01).

There was no significant difference between groups in the epididymal histomorphometry for tail morphology (Fig. 1P) or epithelial height (Fig. 1I; *P* > 0.05). However, regarding the sex glands, the vesicular glands of the hypo rats had less-pleated acini (Fig. 1Q2) and reduced epithelium height compared to control (Fig. 1J; *P < 0.05). Likewise, the prostatic acini of hypothyroid rats also showed a reduced epithelium height compared to control (Fig. 1K and R2; ***P < 0.001). The Kp10 treatment restored the height of the vesicular gland epithelium in hypothyroid rats, matching the control levels (Fig. 1J and Q3; *P* > 0.05), while the height of the prostatic epithelium did not differ in relation to the hypo group (Fig. 1K and R3; *P* > 0.05).

### Hypothyroidism reduces the testicular expression of the Kiss1/Kiss1r system

**Figure 2:**
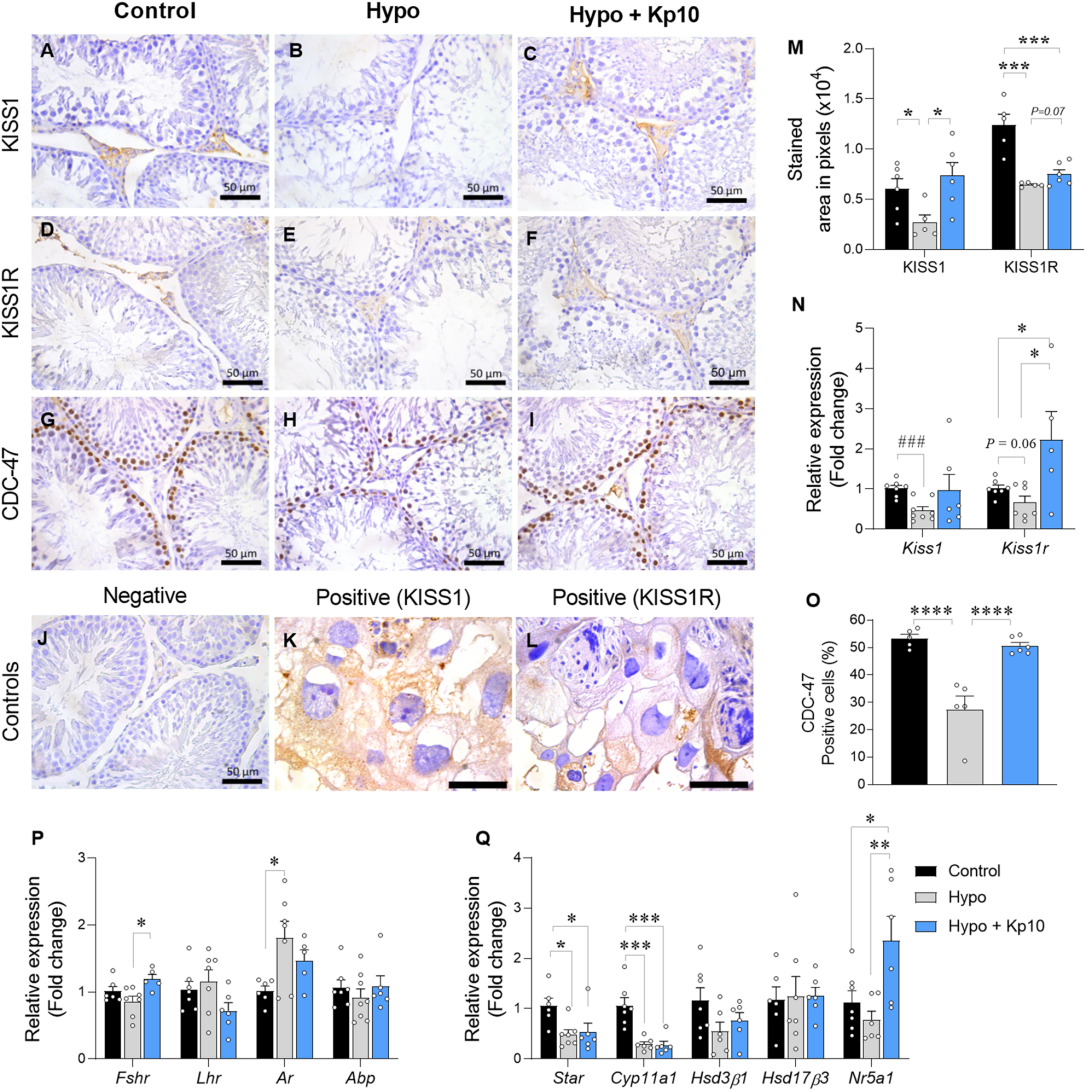
Immunostaining of kiss1/kiss1r and CDC-47 and gene expression of *Kiss1*, *Kiss1r* and hormonal mediators in the testis of control, hypothyroid and Kp10-treated hypothyroid rats. Immunostaining of Kiss1 (**A**–**C**), Kiss1r (**D**–**F**) and CDC-47 (**G**–**I**) in seminiferous tubules of rats from the Control (**A**,**D**,**G**), Hypo (**B**,**E**,**H**) and Hypo + Kp10 (**C**,**F**,**I**) groups. Negative (**J**) and positive (**K**,**L**) controls for immunohistochemical; Bar scale: 50 µm. (**M**) Kiss1 and Kiss1r pixel immunostaining area in the testis of Control, Hypo and Hypo + Kp10 rats; mean ± SEM; **P < 0.01; ***P < 0.001, n = 5–6; (**N**) Relative gene expression (Fold change) of *Kiss1* and *Kiss1r* in the testis of rats from the Control, Hypo and Hypo + Kp10 groups; mean ± SEM; *P < 0.05; n = 5–8; ### = Student t test, *P* < 0.001. (**O**) Percentage of cells positive for CDC-47 in the testis of rats in the Control, Hypo and Hypo + Kp10 groups; mean ± SEM; ****P < 0.0001, n = 5–6; (**P**) Relative expression (Fold change) of *Fshr*, *Lhr*, *Ar* and *Abp* in the testis of Control, Hypo and Hypo + Kp10 rats; mean ± SEM; * *P* < 0.05, n = 6–7; (**Q**) Relative expression (Fold change) of *Star*, *Cyp11a1*, *Hsd3β1*, *Hsd17β3* and *Nr5a1* in the testis of rats in the Control, Hypo and Hypo + Kp10 groups; mean ± SEM; *P < 0.05; **P < 0.01; ***P < 0.001, n = 6–7.

Considering that the Kp10 treatment improved the testicular morphology of the hypothyroid rats, we assessed the Kiss1/Kiss1r system expression in the animals’ testes, as studies suggest the local importance of this system for the male gonadal function^32,33^. Immunostaining analysis of Kiss1 and Kiss1r demonstrated that both have little expression in the rat testis, with expression mainly occurring in the interstitial cells (Fig. 2A–F). There was a reduction in the stained area of Kiss1 (Fig. 2B,M; **P<0.01) and Kiss1r (Fig. 2E,M; ***P < 0.001) in rats with hypothyroidism. Kp10 treatment, in turn, increased the Kiss1 immunostaining area compared to the hypo group (Fig. 2C,M; **P < 0.01), and an increasing trend was also observed for Kiss1r immunostaining relative to the hypo group (Fig. 2F,M; *P* = 0.07). Regarding gene expression, hypo rats also showed a significant reduction in *Kiss1* mRNA levels compared to control (Fig. 2N; ###*P* < 0.001), while there was no difference between the control and the hypo + Kp10 groups (Fig. 2N; *P* > 0.05). Meanwhile, the expression of transcripts for *Kiss1r* showed a tendency towards a reduction in the testes of rats with hypothyroidism compared to control (Fig. 2N; *P* = 0.06). Interestingly, there was also an increase in *Kiss1r* expression in hypo + Kp10 rats relative to the control and hypo groups (Fig. 2N; *P < 0.05).

### Kp10 treatment restores the spermatogonial proliferation in hypothyroid rats

In addition to Kiss1 and Kiss1r expression, we also evaluated the immunostaining of CDC-47, which is an important indicator of cell proliferation. Only cells with densely stained nuclei were considered positive in this analysis. Control rats had approximately 53% of spermatogonia positive for CDC-47 (Fig. 2G,O), while this percentage was significantly reduced in the seminiferous tubules of hypo animals (Fig. 2H,O; ****P < 0.0001). The treatment with Kp10 reestablished the quantity of stained cells (****P < 0.0001), which were similar to the control group (Fig. 2I,O; *P* > 0.05).

### Hypothyroidism affects testicular steroidogenesis and Kp10 treatment increases *Fshr* and *Nr5a1* expression

We also evaluated the expression of some hormone receptors and steroidogenic mediators that are crucial for the testicular function^17,34^. There was no difference between the control and hypo groups for *Fshr* and *Lhr* gene expression, but the Kp10 treatment increased *Fshr* expression compared to the hypo group (Fig. 2P; *P < 0.05). Interestingly, hypo rats showed an increased expression for the *androgen receptor* (*Ar*) mRNA compared to control (Fig. 2P; *P < 0.05), and the animals treated with Kp10 showed no difference compared to the control and hypo groups (Fig. 2P; *P* > 0.05). No difference was observed between groups for the *Abp* expression (Fig. 2P; *P* > 0.05).

Regarding transporter protein and steroidogenic enzyme expression, hypothyroidism reduced *Star* (Fig. 2Q; *P < 0.05) and *Cyp11a1* (Fig. 2Q; ***P < *0.001)* mRNA levels compared to control, and Kp10 did not revert these changes. However, hypo + Kp10 rats showed a greater expression of the transcripts for *Nr5a1* compared to hypo (**P < 0.01) and control (Fig. 2Q; *P< 0.05) groups. No differences were observed between groups for *Hsd3β1* and *Hsd17β3* expression (Fig. 2Q; *P* > 0.05).

### Kp10 treatment improves sperm quality in rats with hypothyroidism

**Figure 3:**
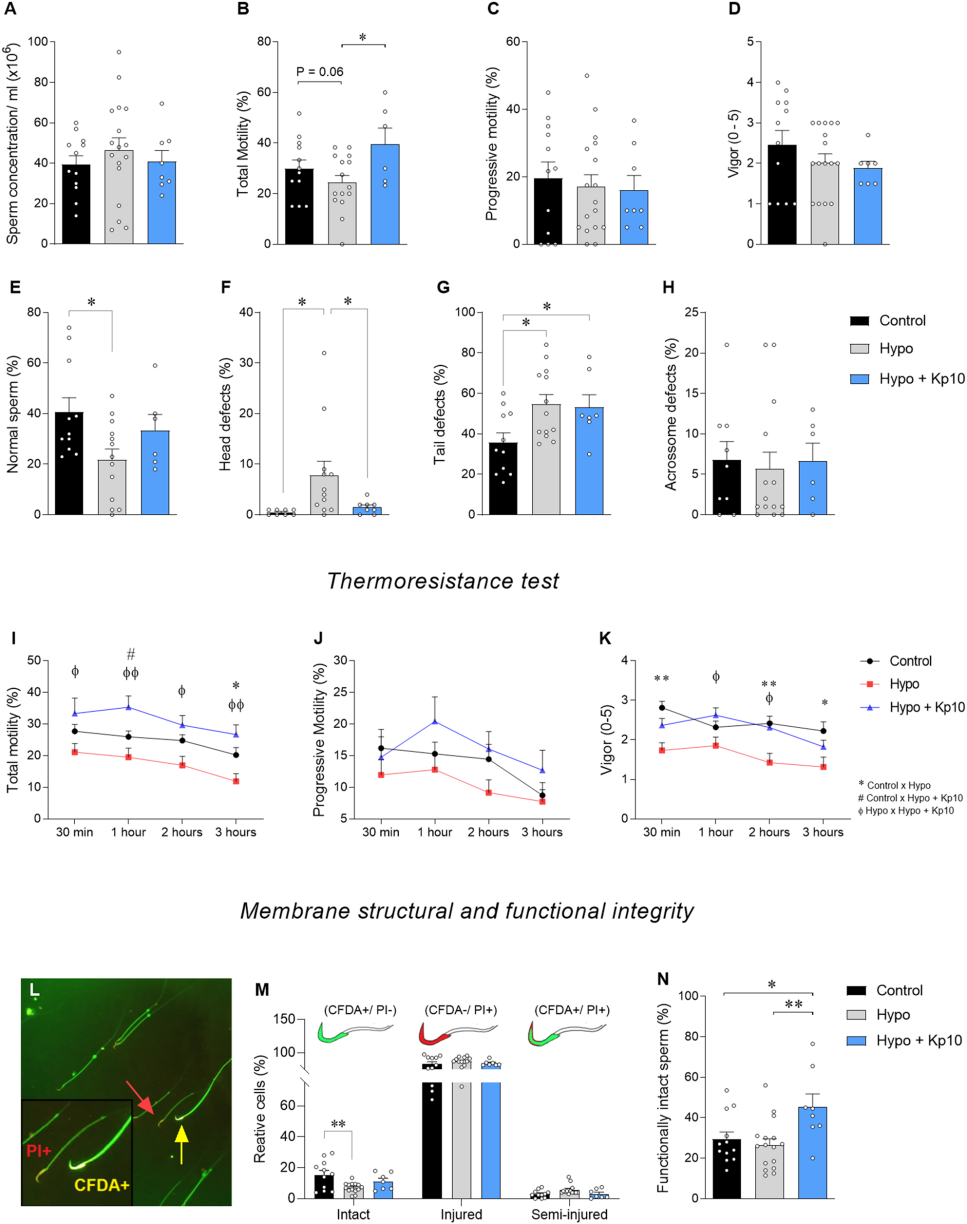
Effects of hypothyroidism and Kp10 treatment on sperm quality in rats. (**A**–**D**) Concentration parameters and sperm kinetics for rats in the Control, Hypo and Hypo + Kp10 groups: (**A**) Sperm concentration (mL × 10^6^); (**B**) Total motility (%); (**C**) Progressive motility (%); and (**D**) Vigor (0–5); mean ± SEM; *P < 0.05; n = 6–17; (**E**–**H**) Sperm morphology for rats in the Control, Hypo and Hypo + Kp10 groups; (E) Normal spermatozoa (%); (**F**) Spermatozoa with head defects (%); (**G**) Sperm with tail defects (%); and spermatozoa with acrosome defects (%); mean ± SEM; *P < 0.05; n = 100 sperm/animal. (**I**–**K**) Thermal resistance test at 30 min, 1, 2 and 3 h in spermatozoa from the Control, Hypo and Hypo + Kp10 groups: (**I**) Total motility (%); (J) Progressive motility (%); and (**K**) Vigor (0–5); mean ± SEM; *P < 0.05; **P < 0.01; # = Student t Test, P < 0.05; n = 8–17 animals/group. (**L**–**N**) Functional and structural test of plasma and acrosomal membranes. (**L**) Immunofluorescence photomicrograph showing intact (CFDA+/PI−) and damaged (CFDA−/PI+) spermatozoa. (**M**) Schematic diagram and quantification of reactive cells (%) for CFDA (intact cells), PI (injured cells) and CFDA/PI (semi-injured cells) of Control, Hypo and Hypo + Kp10 groups; mean ± SEM; *P < 0.05; n = 8–17 animals/group and 200 sperm/animal. (**N**) Hypoosmotic test in sperm from the Control, Hypo and Hypo + Kp10 groups; mean ± SEM; *P < 0.05; **P < 0.01; n = 8–17 animals/group and 200 sperm/animal.

Considering that kisspeptin improved gonadal morphology and increased testicular expression of *Kiss1*, *Kiss1r, Fshr,* and *Nr5a1* in hypothyroid rats and that hypothyroidism reduces sperm quality^35^, we investigated whether Kp10 treatment was able to improve sperm quality in hypothyroid rats. For this purpose, spermatozoa were collected from the tail of the epididymis. No differences were observed between groups in sperm concentration (Fig. 3A; *P* > 0.05), progressive motility (Fig. 3C; *P* > 0.05), and vigor (Fig. 3D; *P*>0.05). However, a trend towards a reduction in total sperm motility was observed in hypo rats relative to control (Fig. 3B; *P* = 0.06), whereas Kp10 treatment increased the sperm motility in hypothyroid rats (Fig. 3B; *P < 0.05).

The analysis of sperm morphology revealed a lower percentage of normal spermatozoa in hypo rats compared to control (Fig. 3E; *P < 0.05) and Kp10 treatment improved this parameter, with no difference from control (Fig. 3E; *P* > 0.05). Hypothyroidism also increased the number of defects in the sperm head (Fig. 3F; *P < 0.05) and tail (Fig. 3G; *P< 0.05), whereas Kp10 treatment was able to reduce the percentage of head defects (Fig. 3F; *P < 0.05), matching the control levels (*P* > 0.05). No differences were observed between groups for acrosome defects (Fig. 3H; *P* > 0.05).

In order to better evaluate the possible cytoprotective effects of Kp10, we performed a heat resistance test and analyzed sperm motility and vigor after 30 min, 1, 2, and 3 h of exposure to heat. Hypothyroidism reduced total motility at 3 h (Fig. 3I; *P < 0.05) and vigor at 30 min, 2 h, and 3 h compared to control (Fig. 3K; *P < 0.05; **P < 0.01). On the other hand, Kp10 treatment resulted in a higher percentage of total sperm motility compared to the hypo group at all time points (Fig. 3I; ^φ^*P* < 0.05; ^φφ^*P* < 0.01), and to control at 1 h (Fig. 3I; #*P* < 0.05). As for vigor, the hypo + Kp10 group also showed an increase in this parameter at 1 and 2 h compared to hypo animals (Fig. 3K; ^φ^*P* < 0.05). No differences in progressive motility were observed between groups (Fig. 3J; *P* > 0.05).

After the sperm heat resistance test, the structural and functional integrity of the plasmatic and acrosomal membranes were evaluated through positive reaction to the CFDA and PI dyes (Fig. 3L). The number of spermatozoa with intact membranes (CFDA+/PI−) was significantly reduced in hypo rats (Fig. 3M; *P < 0.05), while the hypo + Kp10 group showed no difference from the control and hypo groups (Fig. 3M; *P* > 0.05). A similar percentage of cells with injured (CFDA−/PI+) and semi-injured (CFDA+/PI+) acrosomal and plasma membranes was observed between groups (Fig. 3M; *P* > 0.05). In addition, the hypoosmotic test for the plasma membrane functionality showed higher amounts of reactive spermatozoa after the Kp10 treatment compared to the control and hypo groups (Fig. 3N; *P < 0.05; **P < 0.01), indicating that Kp10 treatment improves cell membrane functionality in a hypoosmotic environment.

### Kp10 treatment reverses high PRL levels and increases LH and testosterone secretion in hypothyroid rats

**Figure 4:**
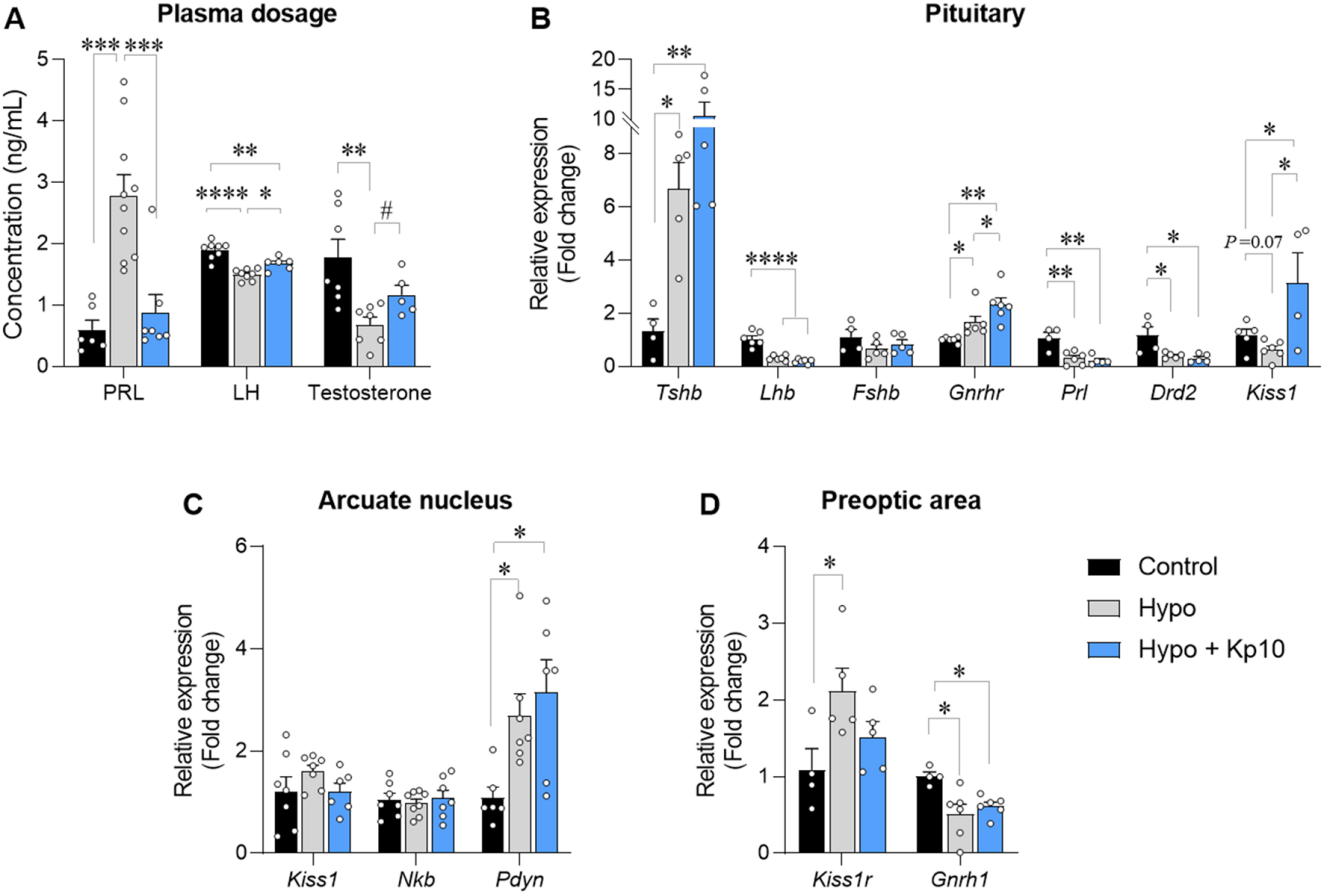
Effects of hypothyroidism and Kp10 treatment on plasma PRL, LH and testosterone levels and on pituitary and hypothalamic expression of genes associated with the HPG and HPT axis. (**A**) Plasma PRL, LH and T levels of rats in the Control, Hypo and Hypo + Kp10 groups (mean ± SEM; *P < 0.05; **P < 0.01; ***P < 0.001; ****P < 0.0001; n = 8; # = Student t Test, *P* < 0.05; (**B**) Relative gene expression of *Tshb*, *Lhb*, *Fshb*, *Gnrhr*, *Plr*, *Drd2* and *Kiss1* in the pituitary of rats in the Control, Hypo and Hypo groups + Kp10 (mean ± SEM; *P < 0.05; **P < 0.01; ****P < 0.0001; n = 4–7); (**C**) Relative gene expression of *Kiss1*, *Nkb* and *Pdyn* in the hypothalamus (ARC) of rats from the Control, Hypo and Hypo + Kp10 groups (mean ± SEM; *P < 0.05; n = 6). (**D**) Relative gene expression of *Kiss1r* and *Gnrh1* in the hypothalamus (APO) of rats from the Control, Hypo and Hypo + Kp10 groups (mean ± SEM; *P < 0.05; n = 4–6).

Given that Kp10 treatment improved testicular and sexual gland morphology and positively affected sperm quality, we evaluated the plasma levels of PRL, LH and testosterone in the animals. Hypothyroidism increased PRL levels fivefold (Fig. 4A; ***P < 0.001) and reduced plasma LH (Fig. 4A; ****P < 0.0001) and testosterone levels (Fig. 4A; **P < 0.01) compared to control. Kp10 treatment, in turn, not only restored plasma PRL (Fig. 4A; ***P < 0.001) and testosterone (Fig. 4A; #*P* < 0.05) to the control levels (P > 0.05) but also increased LH levels (Fig. 4A; *P < 0.05).

### Pituitary gene expression in hypothyroid rats and the effects of Kp10

To better evaluate the HPG axis and considering the hormonal alterations observed in hypo and hypo + Kp10 rats, the pituitary gene expression of *Tshb*, *Lhb, Fshb*, *Gnrhr*, *Prl*, *Drd2,* and *Kiss1* were assessed. Hypothyroidism increased *Tshb* expression (Fig. 4B; *P < *0.05)* and reduced *Lhb, Prl,* and *Drd2* mRNA expression compared to control (Fig. 4B; *P < 0.05; **P < 0.01; ***P < 0.001), and Kp10 treatment did not alter the expression of these genes in hypothyroid rats (Fig. 4B; *P* > 0.05). Hypothyroidism increased *Gnrhr* transcripts relative to control (Fig. 4B; *P < 0.05) and Kp10 further increased its expression relative to both the control and hypothyroid groups (Fig. 4B; *P < 0.05; **P < 0.01). Moreover, a downward trend in *Kiss1* mRNA levels was observed in the pituitary of hypo rats (Fig. 4B; *P* = 0.07), and Kp10 treatment increased *Kiss1* expression compared to the control and hypo groups (Fig. 4B; *P < 0.05). No difference was observed between groups for the *Fshb* expression (Fig. 4B; *P* > 0.05).

### Hypothyroidism increases hypothalamic *Pdyn* and *Kiss1r* gene expression and reduces *Gnrh1* in male rats

Given that there was a reduction in plasma LH and pituitary *Lhb* expression in hypothyroid rats, we evaluated *Kiss1*, *Nkb,* and *pro-dynorphin* (*Pdyn*) gene expression by KNDy neurons in the ARC and *Kiss1r* and *Gnrh1* expression in the POA. We found that neither hypothyroidism nor Kp10 treatment did affected *Kiss1* mRNA in the ARC (Fig. 4C; *P* > 0.05), and the same was observed for *Nkb* expression (Fig. 4C; *P* > 0.05). Nevertheless, the expression of *Pdyn* was higher in hypo rats compared to control (Fig. 4C; *P < 0.05), which was not affected by the Kp10 treatment (Fig. 4C; *P* > 0.05). Hypothyroidism reduced the mRNA levels of *Gnrh1* in the POA compared to control (Fig. 4D; *P < 0.05) while increased *Kiss1r* transcripts (Fig. 4D; *P < 0.05). The Kp10 treatment did not alter the lower expression of *Gnrh1* in hypothyroid animals but partially reversed the increase in *Kiss1r* expression (Fig. 4D; *P* > 0.05).

## Discussion

The present study describes the suppressive effects of hypothyroidism on the gonadal axis of adult male rats, including the impairment of testicular morphology and steroidogenesis, sperm quality and testicular kiss1/kiss1r expression. These changes were accompanied by an increase in plasma PRL levels, a reduction in LH and testosterone, an increase in *Pdyn* in KNDy neurons and a reduction in hypothalamic *Gnrh1*. Treatment with Kp10 reversed the high PRL levels and increased plasma levels of LH and testosterone in hypothyroid animals. Moreover, it improved gonadal morphology and sperm quality and increased the testicular Kiss1/Kiss1r system and *Fshr* and *Nr5a1* expression.

Hypothyroidism induced by PTU administration was confirmed by the reduction in free T_4_, increase in pituitary *Tshb,* and reduction in the animal’s body mass, similar to previous studies where thyroid hypofunction was induced in male rats with PTU^10,11,36^. The effects of PTU can be accounted to hypothyroidism and not to any direct effect, since MMI treatment has similar effects on gonadal function and hormone levels, and T_4_ treatment restored normal functions^12^. Female rats mated with hypothyroid male rats or Kp10-treated hypothyroid rats had a lower birth rate, although no difference was observed in litter size or offspring body mass. This lower birth rate can be explained by the lower percentage of females that copulated with the rats of the hypo and hypo + Kp10 groups and a higher percentage of pregnancy loss, as only 33–50% of the females that copulated had offspring. These data suggest that the reduced birth rate observed in the females paired with hypo and hypo + Kp10 rats results not only from the lower mating rate of the male rats but also from lower sperm quality. Likewise, other studies have demonstrated that hypothyroidism compromises sperm quality^3,4^, and this effect is associated with reduced *in vivo* and *in vitro* fertilization rate^5^. Furthermore, the lower percentage of females that copulated may be the result not only of the lower body mass presented by hypothyroid rats^4,10^, which influences copulation activity in rodents, but also of a lower sexual interest given that studies have already demonstrated a reduced sexual desire in hypothyroid male rats^5,37^.

Hypothyroidism reduced testicular and sex gland mass, decreased seminiferous and glandular epithelium height, and caused testicular degeneration. These results are consistent with previous studies in hypothyroid rats that also described changes in testicular morphology^4,11,12,38^ and a reduction in the mass of genital tract organs^10,39–43^. Kp10 not only increased the mass of the vesicular and prostate gland but also restored the height of the seminiferous epithelium and vesicular gland. The increases in sex gland mass and vesicular gland epithelium height probably reflect the reestablishment of testosterone levels caused by Kp10 in hypothyroid rats, given that hypothyroidism reduced testosterone that has a trophic effect on the sex glands^44^. Furthermore, it is plausible to speculate that the reestablishment of the seminiferous epithelium height after Kp10 treatment resulted not only from the increase in the proliferative activity of spermatogonia, evidenced by the CDC-47 immunostaining, but also from the increase in testicular *Fshr* expression, as FSH is the key hormone in the seminiferous epithelium proliferation^45^.

In addition to the macroscopic and histological alterations, hypothyroidism reduced Kiss1 and Kiss1r immunostaining and gene expression in the rat’s testis. Kp10 treatment was able to restore Kiss1 protein levels and *Kiss1r* gene expression*,* with *Kiss1r* expression being even greater than in the control. This suggests a positive self-regulation of the Kiss1/Kiss1r system in this organ. Furthermore, both were mainly localized in the interstitium, similar to previous studies in mice, goats, horses, and non-human primates^27–30^, which reinforces a role for the local kiss/kiss1r system in testicular steroidogenesis^23^. Accordingly, a previous study using Leydig cells of goats demonstrated that kisspeptin stimulates testosterone production and *Kiss1* and *Kiss1r* gene expression in these cells^29^.

When we evaluated the expression of key enzymes in the steroidogenic cascade, hypothyroidism reduced testicular *Star* and *Cyp11a1* expression*.* This explains the lower testosterone levels observed in these animals, which has also been demonstrated in other studies^38,43,46^. While Kp10 treatment did not reestablish the change in *Star* and *Cyp11a1* expression caused by hypothyroidism, it increased nuclear receptor subfamily 5 group A member 1 (*Nr5a1*), also named steroidogenic factor 1, which is an important transcription factor for testicular steroidogenesis^47^. Therefore, the increase in *Nr5a1* may be associated with the recovery of testosterone concentration observed in hypo + Kp10 rats. This increase in testosterone may have also been influenced by the partial restoration of LH secretion observed in these animals, as LH acts on Leydig cells to stimulate steroidogenesis^48^.

As for hormone receptors and membrane transporters, hypothyroidism increased *Ar* expression in the testes. Similar to the present study, Chang et al.^49^ observed an increase in testicular *Ar* mRNA in hypothyroid rats but a reduction in the AR protein levels. This suggests that there are post-transcriptional alterations in testicular AR expression in hypothyroid rats^49^. Furthermore, the increase in *Ar* mRNA observed in hypo rats may be the result of the reduction in plasma testosterone, because androgens negatively regulat*e Ar* gene expression^50,51^.

Assessment of sperm parameters showed that hypothyroidism reduced total motility, increased head and tail defects, and reduced sperm structural integrity. These results are in line with previous studies of hypothyroidism that described changes in sperm morphology in rodents and men^6,52^, and in sperm kinetics in sheep and rats^38,53,54^. Kp10, in turn, increased total motility, reduced head defects, increased the number of spermatozoa with intact and functional cell membranes, and, surprisingly, avoided the reduction in thermoresistance caused by hypothyroidism. In fact, total motility was even greater than in the control at 1 h in the heat tolerance test. Different mechanisms can be suggested to explain the improved sperm quality in hypothyroid rats caused by Kp10. First, the observed improvement in testicular morphology, which provides a suitable environment for spermatogenesis. Second, a direct effect of Kp10 on the sperm, consistent with the report that kisspeptin increases sperm motility *in vitro*^55^. Furthermore, kisspeptin showed an antioxidant action in models of ovarian and uterine ischemia^56^, lipid peroxidation in the testis^57^, and placental oxidative stress caused by hypothyroidism^58^. Thus, Kp10 may have protected against the oxidative stress caused by hypothyroidism in the testis^4,59^, although more studies are needed to prove this hypothesis. It is important to point out that the Kiss1/Kiss1r system is also expressed in the epididymis of rats and Kiss1r expression in the spermatozoa changes during the epididymal transit, suggesting the participation of this system in sperm maturation^60,61^. Thus, changes in the epididymis itself may also have favored the improvement in sperm quality after the Kp10 treatment. However, more studies are needed to elucidate the role of kisspeptin in the spermatogenesis.

Hypothyroid rats exhibited an increase in PRL in addition to the reduction in LH and testosterone, which are well-known effects of this disorder^10,11,38^. Kp10 not only increased testosterone and LH levels but also blocked the increase in plasma PRL caused by hypothyroidism. This result is surprising considering that previous studies have shown that central kisspeptin administration stimulates PRL secretion in rats and mice^62,63^. Although the PRL values in the present study are low, other studies with male rats have shown discrepancy in the range of PRL levels^64–69^, which may be the result of different ELISA protocols and, mainly, different models of hyperprolactinemia (for example: induction by administration of ovine PRL, cabergoline, haloperidol and others, in addition to surgical methods). Notably, the effect of Kp10 on PRL concentration observed here in the male was different from that recently described in hypothyroid female^20^, in which Kp10 increased plasma LH and restored ovarian cyclicity without altering the hyperprolactinemia^20^. This suggests that there are differences in the PRL modulation by kisspeptin between male and female rats in the hypothyroid condition. Despite the increase in plasma PRL in hypothyroid rats, there was a reduction in pituitary *Prl* expression, which may be a result of the negative-feedback effect of PRL on its own secretion at the transcriptional level^70^.

In the present study, hypothyroidism also reduced *Drd2* gene expression in the pituitary, likewise in hypothyroid female rats^20^. This suggests another mechanism through which hypothyroidism can cause hyperprolactinemia in addition to the recognized stimulatory effect of the increased TRH on PRL secretion during hypothyroidism^71^. Kp10 treatment, on the other hand, had no effect on the change in *Drd2* expression caused by hypothyroidism, which suggests that this pathway is not involved in the PRL suppression caused by Kp10 in hypothyroid male rats. Navarro et al.^72^ demonstrated that the intracerebroventricular administration of Kp10 decreased PRL levels in prepubertal male rats, but no effect was seen in adult male rats. However, previous studies have demonstrated a stimulatory effect of kisspeptin on PRL secretion in adult male rats^62^ and an inhibitory effect on dopamine secretion from Hypo-E22 hypothalamic rat cells^73^. Thus, although the mechanism is presently unclear, the effect of kisspeptin on PRL secretion seems to vary with the thyroid status and sex.

Despite the increase in the plasma LH levels, Kp10 treatment did not reverse the lower *Lhb* gene expression caused by hypothyroidism, unlike what was observed in Kp10-treated hypothyroid female rats^20^. This result suggests that the increase in plasma LH caused by Kp10 was caused by changes at the post-transcriptional level in the gonadotrophs. Furthermore, the lower *Lhb* expression observed in hypothyroid rats was associated with the reduced *Gnrh1* expression in the hypothalamus, suggesting reduced synthesis and therefore release of GnRH in the portal blood^74^. Of note, despite the lower *Lhb* and *Gnrh1* expression, rats in the hypothyroid and Kp10-treated hypothyroid groups exhibited an increased pituitary *Gnrhr* expression. This increase in *Gnrhr* may have resulted from the biphasic regulation of pituitary GnRHr by GnRH, with stimulation by low concentrations of GnRH and inhibition by high levels of this hormone^75^. Since hypothyroid rats had lower hypothalamic *Gnrh1* expression, it is plausible to suppose that they had lower but not absent GnRH release, which might have increased the expression of *Gnrhr* in the gonadotrophs. Moreover, Kp10 treatment further increased the *Gnrhr* expression compared to the hypothyroid group, which may reflect the direct action of Kp10 on the gonadotrophs, since kisspeptin administration stimulates GnRHr expression in the GnRH-producing GT1-7 hypothalamic cells that overexpress the GRP54 receptor^76^. It is noteworthy that Kp10 administration also increased the pituitary *Kiss1* expression. This effect may have contributed not only to the greater pituitary *Gnrhr* expression^76^ but also favored the higher LH levels observed in Kp10-treated animals, since kisspeptin increases LH expression in the mouse pituitary and gonadotropic Lβt2 cells^77^.

We did not observe changes in *Kiss1* or *Nkb* in the ARC of hypothyroid or Kp10-treated hypothyroid rats. Unlike what was observed in the ARC of female rats with hypothyroidism, which showed a reduction in Kiss1 protein and mRNA and *Nkb* mRNA^20^. However, in the present study, hypothyroidism increased *Pdyn* expression in the ARC, which is consistent with the reduction in *Gnrh1* expression in the POA of these animals. Dynorphin, whose precursor is Pdyn, is known for its role in negatively regulating kisspeptin secretion in the gonadotrophic axis and, consequently, the pulsatility of GnRH/LH^78–80^. Furthermore, dynorphin inhibits TIDA neuron activity^79,80^, which suggests another pathway by which hypothyroid male rats exhibit high PRL levels. The results of the present study suggest that the negative impact of hypothyroidism on the HPG axis of male rats differs from that in female rats, since no difference in *Pdyn* expression was observed in the females^20^. Furthermore, curiously, an increase in *Kiss1r* gene expression was observed in the POA of hypothyroid animals, while the Kp10-treated group showed no difference in this parameter. The increase in *Kiss1r* caused by hypothyroidism may be a compensatory effect of the reduced hypothalamic *Gnrh1* expression, although further studies are needed to elucidate this issue.

The findings of the present study describe the inhibitory effects of hypothyroidism on the HPG axis of male rats, with inhibition of the testicular Kiss1/Kiss1r pathway and an increase in hypothalamic *Pdyn* expression. Kp10 treatment improves gonadal and sex gland morphology, LH and testosterone levels, and sperm quality. Kp10 also blocks the increase in plasma PRL caused by hypothyroidism. This suggests that kisspeptin analogs may be promising in the treatment of male reproductive dysfunctions caused by thyroid hypofunction.

## Materials and methods

### Animals

Three-month-old male Wistar rats (371.3 ± 3.81 g) were used, obtained from the Animal Breeding, Maintenance and Experimentation Laboratory of the Universidade Estadual de Santa Cruz (UESC). The animals were housed in plastic boxes (6 animals/box) with controlled temperature (22 ± 2 °C), light (1200 h light/1200 h dark), and airflow and with water and commercial feed provided *ad libitum*. All experimental procedures were approved by the Ethics Committee on Animal Use (CEUA) of the UESC (Protocol No. 03/19) and were performed according to the ARRIVE guidelines (Animal Research: Reporting of *In Vivo* Experiments—https://arriveguidelines.org/) and the International Council for Laboratory Animal Science (ICLAS).

### Experimental design

The rats were randomly assigned into control (n = 15), hypothyroid (hypo; n = 17), and Kp10-treated hypothyroid (hypo + Kp10; n = 14) groups. The hypothyroid groups received a daily dose of 4 mg/kg/day of 6-propyl-2-thiouracil (PTU)^81^, diluted in 3 mL of distilled water, via orogastric tube, while the control group received water as a placebo. After 60 days of treatment, the rats in the Hypo+Kp10 group began to receive daily Kp10 (12 µg/kg/day; Cat. No. 4243, Tocris Bioscience, Bristol, UK) via intraperitoneal (i.p.) administration. After three months of PTU administration, all animals were euthanized between 10:00 and 11:00 h by decapitation. Blood samples were taken to quantify free thyroxine (T_4_), thyroid-stimulating hormone (TSH), prolactin (PRL), LH and testosterone by enzyme-linked immunosorbent assay (ELISA). The genital tract was removed and the testis, epididymis, vesicular gland, and prostate were collected for morphological and/or immunohistochemical (IHC) analyses. The tail of one of the epididymis was also collected and immediately processed for sperm assessment. Hypothalamus, pituitary, and testis samples were also collected, immersed in TRIzol, immediately frozen in liquid nitrogen and stored in a freezer at − 80 °C for gene expression by real-time polymerase chain reaction (RT-qPCR).

### Hormone dosage

Blood was collected in tubes with heparin and the samples were centrifuged at 3000 rpm for 20 min at 4 °C to obtain the plasma, which was stored at –20 °C until the moment of hormonal analysis. Assessment of free T_4_ was performed by ELISA following the manufacturer's recommendations (IMMULITE, Siemens Medical Solutions Diagnostics, Malvern, PA, USA), and plasma LH and PRL levels were assessed by ultrasensitive ELISA, according to Aquino et al.^82^. For these assays, the reference preparation used as the standard curve for PRL were rat PRL (rPRL) RP-3 [AFP-4459B, National Institute of Diabetes and Digestive and Kidney Diseases-National Hormone and Pituitary Program (NIDDK-NHPP)] and LH mouse (rLH) RP-3 (AFP718B, NIDDK-NHPP)^82^.

### Tissue collection, histological processing and testicular and glandular histomorphometry

The testis, epididymis, prostate, and seminal vesicle were fixed in 4% paraformaldehyde for 24 h and 1.5% glutaraldehyde was added to the fixative for the histological analysis of the testis. The tissues were dehydrated in a gradual solution from 70% ethyl alcohol to absolute alcohol, cleared in xylene, and embedded in paraffin. Histological sections of 4 µm were obtained by microtomy on common and silanized slides (StarFrost Polycat, Germany) to perform histomorphometry and IHC, respectively.

#### Testicular histomorphometry

Testicular histomorphometry was performed based on Lara and França^83^ with adaptations. In short, the tubular diameter and height of the epithelium were obtained using the Image-Pro Plus software at 200× magnification. Thirty round or nearly round tubules per animal were randomly evaluated in five histological sections spaced by 10 µm. The tubular diameter values were obtained from an average of two vertically and horizontally perpendicular measurements in each tubule, while average values of epithelial height were obtained from four equidistant measurements in each tubule.

#### Epididymal and glandular histomorphometry

In the seminal vesicle and prostate, the epithelial height was measured at four equidistant points per glandular acinus, obtaining an average of 40 to 50 glandular acini per animal. In the epididymis tail epithelium, four equidistant points were also measured per tubule, with a mean obtained from 40 to 50 tubular regions per animal.

### Mating test

To test reproductive function, male rats from the control, hypo and hypo + Kp10 groups were individually housed with 3-month-old female rats for a maximum period of nine days before euthanasia, which corresponds to approximately two estrous cycles for female rats. Before the mating period, we checked the cyclicity of the females and they had regular cycles (Supplement Fig. S1 A–C).

To carry out this experiment, two mating groups were formed. In the first, eight male rats per group were used and the females separated after nine days. No manipulation was performed on these animals, such as vaginal smears, in order to avoid stress and possible impacts on gestational success. For these animals, only the parameters of number of natural births and litter size were evaluated.

In the second group, seven, five, and six male rats from the control, hypo and hypo + Kp10 groups were mated, respectively. In this experiment, females had vaginal cytology monitored daily between 7:00 am and 8:00 am and the presence of sperm in the smear characterized zero day of pregnancy (0 DG). Thus, in addition to the total number of natural births and litter size, we quantified the number of females that had copulation confirmed by vaginal cytology and estimated a percentage of pregnancy loss. The percentage of females with gestational loss was calculated based on the number of females that gave birth related to the number of females that copulated.

### Sperm analysis

The tail of the right epididymis was cut in a petri dish to obtain sperm-rich epididymal fluid. The fluid was diluted in 500 µL of Tris-citrate-fructose (3.025 g of Tris, 1.7 g of citric acid, 1.25 g of fructose and 100 mL of distilled water) at 37 °C. Aliquots were taken for analysis of sperm morphology, motility and kinetics, and evaluation of the structural and functional integrity of the plasma and acrosomal membranes.

#### Sperm morphology

A 50-µL aliquot of the fluid was fixed in 100 µL of buffered 4 % paraformaldehyde. One hundred spermatozoa were analyzed using a phase contrast microscope at 1000-× magnification and the morphology was classified by total number of acrosome, head and tail defects, according to Couto-Santos et al.^84^. The results were expressed as a percentage of what(?).

#### Motility and kinematics

Sperm motility and kinematics were assessed using a computerized semen analysis system (Sperm Class Analyzer-Automatic system of sperm analysis by computer, MICROPTIC S.L.©, Barcelona, Spain). The analyzed parameters were total motility (MT, %) and progressive motility (MP, %). The vigor was determined by two blind observers, and each sample rated on a scale of 0–5, where “0” indicates cells without movement, “1” vibrating cells, but without locomotion; “2” cells begin progressive movement; “3” indicate moderate movement speed; “4–5” high movement speed.

#### Structural integrity of plasma and acrosomal membranes

The structural integrity of the sperm’s plasma and acrosomal membranes was assessed by the fluorescence technique using two fluorochromes, carboxyfluorescein diacetate (CFDA) and propidium iodide (PI), according to Harrison and Vickers’s^85^ protocol. To prepare the solution, 10 μL of epididymal fluid was added to 40 μL of working solution (20 μL of CFDA, 10 μL of PI, 10 μL of 40% formalin, 960 μL of sodium citrate) and the final solution was incubated for 8 min in a water bath at 37 °C. The spermatozoa were evaluated in an epifluorescence microscope (Nikon, Eclipse 80i) at a 400-× magnification. Two hundred spermatozoa were evaluated, considering three categories: intact (intact plasma and acrosomal membranes; CFDA+/PI−), damaged (damaged plasma and acrosomal membranes; CFDA−/PI+) and semi-damaged (intact plasma membrane and damaged acrosomal membrane; CFDA+/PI+). The result was expressed as a percentage of total spermatozoa.

#### Functional integrity of the plasma membrane

The functional integrity of the plasma membrane was evaluated by the hypoosmotic test^86^. For the hypoosmotic solution, sodium citrate (7.35 g), fructose (13.5 g) and distilled water (1000 mL) were used. The solution was stored at 4 °C until use. Then, 0.1 mL of epididymal fluid was added to 1 mL of hypoosmotic solution and incubated at 37 °C for 1 h. One hundred spermatozoa were analyzed under a phase contrast microscope with 400-× magnification, and the result was expressed as a percentage of cells that were reactive to the test. Reactive spermatozoa are characterized by a swelling of the head and evident folding of the tail, indicating cellular adaptation to the incubation environment.

#### Sperm heat resistance test

Aliquots of epididymal fluid in tris-citrate-fructose diluting solution were kept in a dry bath at 37 °C for 30 min, 1, 2, and 3 h and were once again assessed for total and progressive motility parameters and vigor.

### Immunohistochemistry (IHC)

The antibodies used for the IHC of the testes were anti-KISS1 (1:50, sc-101246, Santa Cruz Biotechnology, CA, USA), anti-KISS1R (1:50, HPA071913, Sigma-Aldrich, Missouri, USA) and anti-MCM7/CDC-47 (1:800, sc-9966, Santa Cruz Biotechnology, CA, USA) to evaluate cell proliferation. All the antibodies used have been previously validated by the manufacturers and used in previous studies^87,88^.

The streptavidin-biotin-peroxidase technique was used by the Dako detection system (EnVision™ FLEX+, Mouse, High pH, (Link)), following the protocol proposed by Ilie et al.^89^ with adaptations. Sections were deparaffinized in xylene, hydrated in a gradual alcohol solution and the epitope was recovered by heat in a citric acid solution (0.54 mol/L; 98 °C; pH 6.0). The sections were immersed for 30 min in a hydrogen peroxide solution (3%; H_2_O_2_) in methanol (CH_3_OH) to block endogenous peroxidase, then kept for another 30 min in a blocking serum solution (Ultra vision Block, Lab Vision Corp., Fremont, CA. USA). Afterward, the sections were incubated for 42 h with the primary antibody in a humid chamber at 4 °C. After washing in buffered saline solution with Tris + Tween 20 (0.05%) (TBS-T; pH 7.6), a protein stabilization solution (EnVision™ FLEX +, Mouse (LINKER)) was added to the sections, followed by the secondary antibody conjugated with streptavidin peroxidase (EnVision™ FLEX/HRP) for 30 min. The chromogen used was 3'3 diaminobenzidine (EnVision™ FLEX DAB+ Chromogen), diluted in buffer with H_2_O_2_ (EnVision™ FLEX Substrate Buffer; 1:50). Sections were counterstained with Harris hematoxylin and negative control was obtained by replacing the primary antibody with TBS-T (Fig. 2J). Rat placenta was used as a positive control for Kiss1 (Fig. 2K) and Kiss1r (Fig. 2L)^88^.

Descriptive and quantitative assessments of Kiss1 and Kiss1R immunostaining were performed on the testis. The immunostaining area was determined using the WCIF ImageJ® software (Media Cybernetics Manufacturing, Rockville, MD, USA) with random photomicrographs taken in 15 regions of the testis by a Leica DM 2500 microscope using the Leica DFC 295 digital camera (Leica Microsystems, Germany). Color deconvolution and thresholding of the images were performed for analysis. Data from each tissue were recorded, analyzed and expressed as immunostaining area in pixels^90^. Quantitative assessment of cell division control protein 47 (CDC-47) was performed on two histological sections/animal to determine the percentage of positive spermatogonia in 30 random seminiferous tubules per animal. Data were expressed as percentage of positive cells.

### Real-time qPCR (RT-qPCR)

The preoptic area (POA) and ARC of the hypothalamus were dissected using a 2 mm punch in coronary sections of the brain performed with a cryostat, according to the rat brain atlas^91^. The APO was dissected at approximately between +0.5 and − 0.5 mm bregma, while for the ARC, it was between − 1.8 and − 3.8 mm bregma^91^. For the real-time RT-qPCR, total RNA was extracted from the hypothalamus, pituitary and testis using TRIzol (Invitrogen, Life Technologies, Carlsbad, CA, USA) as previously described^20^.

For the pituitary and hypothalamus, cDNA synthesis was performed with the SuperScript^TM^ IV First-Strand Synthesis kit (Invitrogen), using 1 µg of RNA. Transcripts of target genes were quantified by qPCR using SYBR Green (Platinum SYBR Green qPCR SuperMix-UDG; Invitrogen) and PowerUp^TM^ SYBR^TM^ Green Master Mix (Invitrogen) for pituitary and hypothalamus, respectively. Amplification was performed on an Applied Biosystems 7500 Fast Real-Time PCR System (Applied Biosystems, Life Technologies). The FAST-cycling conditions for pituitary were initial denaturation at 94 °C for 2 min, 35 cycles of denaturation at 94 °C for 15 s, annealing at 55 °C for 30 s and extension at 68 °C for 1 min. For the hypothalamus, the conditions were enzymatic activation at 50 °C for 2 min, dual-lock DNA polymerase at 95 °C for 2 min, 40 denaturation cycles at 95 °C for 3 s and annealing/extension at 60 °C for 30 s. In the testis, in turn, cDNA synthesis was performed with the GoTaq® 2-Step RT-qPCR System kit (A6010, PROMEGA, Madison, WI, USA), also using 1 µg of RNA. The FAST-cycling conditions in the same apparatus were one cycle of GoTaq® DNA Polymerase activation at 95 °C for 2 min, 40 cycles of denaturation at 95 °C for 3 s and 40 cycles of annealing/extension at 60 °C for 30 s. As negative controls, a complete DNA amplification mix was used, in which the target cDNA template was replaced with UltraPure™ DNase/RNase-Free distilled water (10977015, Thermo Fisher Scientific, Massachusetts, USA). To assess the linearity and efficiency of qPCR amplification, standard curves for all transcripts were generated using serial dilutions of cDNA. A melting curve was obtained for the amplification products to determine their melting temperatures. Primers were designed based on the *Rattus norvegicus* mRNA sequence (Table 1) and the relative gene expression was calculated by the 2^−∆∆CT^ method with calibration based on glyceraldehyde-3-phosphate dehydrogenase (*Gapdh*).

**Table 1:** List of primer pairs for RT-qPCR.

Gene		Forward sequence (5′– > 3′)	Reverse sequence (5′– > 3′)	Accession no.
Gonadotropin releasing hormone 1	*Gnrh1*	TGGTATCCCTTTGGCTTTCACA	TGATCCTCCTCCTTGCCCAT	NM_012767.2
Tachykinin precursor 3	*Nkb/Tac3*	ATAGGCCAGCAGTGCAGAAA	AGCCAACAGGAGGACCTTG	NM_019162.2
Prodynorphin	*Pdyn*	ATGGGGATCAGGTAGGGCAT	ACCGAGTCACCACCTTGAAC	NM_019374.3
KiSS-1 metastasis-suppressor	*Kiss1*	GAGCCACTGGCAAAAATGGC	ATTAACGAGTTCCTGGGGTCC	NM_181692.1
KISS1 receptor	*Gpr54*	CAACCTGCTGGCCCTATACC	TGCAGGGCGCCATCAGT	NM_023992.2
Gonadotropin releasing hormone receptor	*Gnrhr*	CAGCGCTTTGCGTTCAGTTA	CAAGGACGGGCTTCAAGAGT	NM_031038.3
Luteinizing hormone subunit beta	*Lhb*	AGAATGGAGAGGCTCCAGGG	GCAGACTGGGCAGAACTCAT	NM_012858.2
Thyroid stimulating hormone subunit beta	*Tshb*	GTTGGTTTTGACAGCCTCGT	GGCAAACTGTTTCTTCCCAA	NM_013116.2
Follicle stimulating hormone subunit beta	*Fshb*	CCAGCATGATTGCAAGCGAA	AGCCAGCTACGTCAGCATTT	NM_001007597.2
Prolactin	*Plr*	CATCAATGACTGCCCCACTTC	CCAAACTGAGGATCAGGTTCAAA	NM_012629.1
Dopamine receptor D2	*Drd2*	TGCCCTTCATCGTCACTCTG	GGGTACAGTTGCCCTTGAGT	NM_012547.3
Androgen receptor	*Ar*	CAGGGACCACGTTTTACCCA	TTTCCGGAGACGACACGATG	NM_012502.2
Hydroxysteroid (17-beta) dehydrogenase 3	*Hsd17β3*	CAAACTCATCGGCGGTCTTG	TCCAGGTGCTGACCCCTTAT	NM_054007.1
Hydroxy-delta-5-steroid dehydrogenase, 3 beta- and steroid delta-isomerase 1	*Hsd3β1*	AACTGCCACTTGGTCACACTGTC	GTCCGGATCCACTCCGAGGTT	NM_001007719.3
Steroidogenic acute regulatory protein	*Star*	ACCAAGCGTAGAGGTTCCAC	AGCTCTGATGACACCGCTTT	NM_031558.3
Sex hormone binding globulin	*ABP/Shbg*	CGACTGCTTCTGTTGCTA	CAATGTGTCTCAGGGTCCGT	NM_012650.2
Follicle stimulating hormone receptor	*Fshr*	CAAGAACTTCCGCAGGGACT	ATGGAAGTTGTGGGTAGCGG	NM_199237.2
Luteinizing hormone receptor	*Lhr*	CCTGACGGTTATCACCCTGG	GGATGGCATGCCTCAGTCTT	NM_012978.2
Cytochrome P450, family 11, subfamily a, polypeptide 1	*Cyp11a1*	GCAGTCGTGGGGACAGTATG	GGTTGGCAGCTTTTGACCAG	NM_017286.3
Nuclear receptor subfamily 5, group A, member 1	*Nr5a1*	GAAGTTCCTGACAGCCCGATA	CGGATAAGGGTGGACACTGG	NM_001191099.1
Glyceraldehyde-3-phosphate dehydrogenase	*Gapdh*	ACAGCCGCATCTTCTTGTGC	GCCTCACCCCATTTGATGTT	NM_017008.4

### Statistical analysis

Data are presented as mean ± standard error of the mean (SEM). The differences among groups were determined by analysis of variance (ANOVA) followed by the Student-Newman-Keuls test (SNK) for comparison between three experimental groups using the GraphPad Prism® 8.0.2 program. Body mass was assessed over the experimental period by verifying the interaction of factors using two-way ANOVA, followed by multiple comparisons using SNK. All data (masses, spermogram, gene expression and protein quantification) were analyzed for normality of residuals using the Shapiro-Wilk, D'Agostino & Pearson and Kolmogorov-Smirnov tests. When the data did not meet the requirements, logarithmic transformation was performed. The proportion data obtained in the mating tests were compared using the Fisher's exact test. For all analyses, differences were considered significant when *P* < 0.05.

### Supplementary Information

Supplementary Information.

## Data Availability

The data that support the findings of this study are available from the corresponding author, JFS, upon reasonable request.
