# Supplementary material for: Kisspeptin treatment reverses high prolactin levels and improves gonadal function in hypothyroid male rats
Source: Sci Rep. 2023 Oct 5;13:16819. doi: 10.1038/s41598-023-44056-z (PMC10556046; doi:10.1038/s41598-023-44056-z)
Supplement: Supplementary file 1 — Supplementary Information. [file 41598_2023_44056_MOESM1_ESM.pdf]

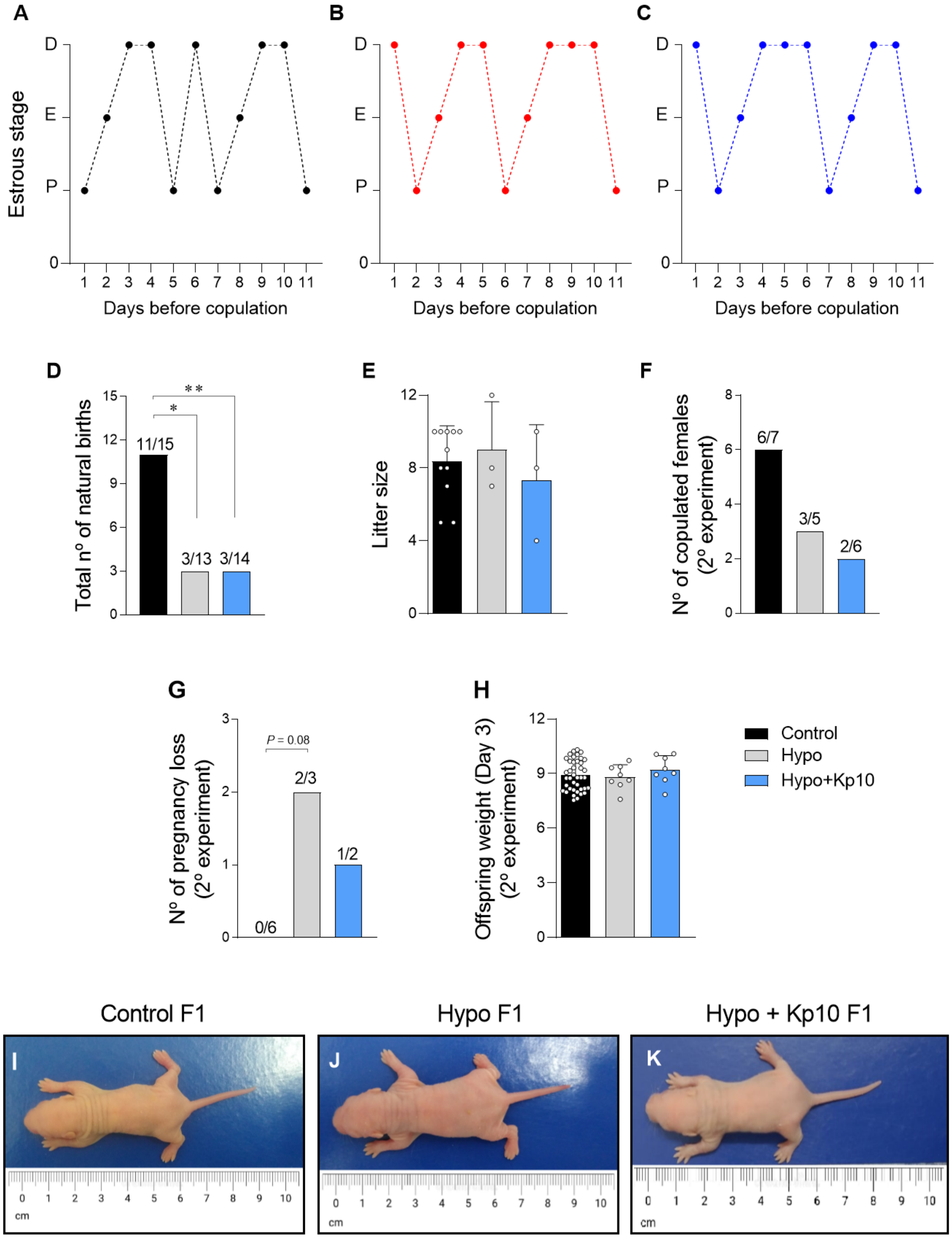

Supplementary Figure 1. Effects of hypothyroidism and Kp10 treatment on the mating of male rats. (A-C) Representative data of evaluation of the estrous cycle of female rats mated with male rats from the control (A), hypo (B) and Hypo + Kp10 (C) groups in the eleven days preceding the mating period; (D) Total number of births of female rats mated with male rats from the Control, Hypo and Hypo + Kp10 groups; (E) Litter size of female rats mated with male rats from the Control, Hypo and Hypo + Kp10 groups; (F) Number of female rats with confirmed copulations with male rats from the Control, Hypo and Hypo + Kp10 groups; (G) Number of female rats that presented embryonic/fetal loss after mating with male rats from the Control, Hypo and Hypo + Kp10 groups; (H) Body mass of pups on the third postnatal day from female rats mated with male rats from the Control, Hypo and Hypo + Kp10 groups; (I-K) Images of offspring on the third postnatal day of female rats mated with male rats from the Control (I), Hypo (J) and Hypo + Kp10 (K) groups; Bar scale: 1 cm.
